# Patient Blood Management in Total Hip and Knee Arthroplasty Before, During, and After the COVID-19 Pandemic: A Single-Center Retrospective Cohort Study

**DOI:** 10.3390/medicina62050942

**Published:** 2026-05-12

**Authors:** Alessandra De Angelis, Marco Minelli, Federico Della Rocca, Enrico Pagot, Federica Martorelli, Vincenzo Simili, Cinzia Elisabetta De Grandis, Marco Scardino

**Affiliations:** 1 IRCCS Humanitas Research Hospital, Via Manzoni 56, 20089 Rozzano, Italy; alessandra.deangelis@humanitas.it (A.D.A.); federico.della_rocca@humanitas.it (F.D.R.); enrico.pagot@humanitas.it (E.P.); federica.martorelli@humanitas.it (F.M.); vincenzo.simili@humanitas.it (V.S.); cinzia.degrandis@humanitas.it (C.E.D.G.); marco.scardino@humanitas.it (M.S.); 2 Department of Biomedical Sciences, Humanitas University, Via Rita Levi Montalcini 4, 20072 Pieve Emanuele, Milan, Italy

**Keywords:** patient blood management, total hip arthroplasty, total knee arthroplasty, COVID-19, blood transfusion

## Abstract

*Background and Objectives*: Patient blood management (PBM) strategies are effective in reducing transfusion requirements and improving perioperative outcomes in total hip arthroplasty (THA) and total knee arthroplasty (TKA). The SARS-CoV-2 pandemic profoundly disrupted hospital organization and limited the application of established PBM protocols. This study assessed the impact of COVID-19-related organizational disruption on PBM implementation and perioperative outcomes in primary total hip and knee arthroplasty. *Materials and Methods*: This monocentric retrospective study included consecutive patients undergoing primary unilateral THA or TKA between January 2019 and February 2023. Patients were stratified into three periods: pre-COVID-19, during COVID-19, and post-COVID-19. Primary outcomes included transfusion rate, allocated RBC units, PRM transfer, hospital length of stay, and postoperative day-4 hemoglobin levels. Secondary outcomes were factors associated with postoperative day-4 hemoglobin and PRM admission. *Results*: A total of 5789 patients were included: 1889 in the pre-COVID-19 period, 1416 during COVID-19, and 2484 post-COVID-19. Despite reduced PBM implementation during the COVID-19 period, postoperative Hb levels and transfusion rates remained stable across groups. The number of allocated red blood cell units increased without a corresponding rise in transfusion rates, suggesting a precautionary allocation strategy. A descriptive reduction in median length of stay was observed in the post-COVID-19 phase. A significant decrease in the proportion of patients transferred to Physical and Rehabilitation Medicine departments during the COVID-19 and in the post-COVID-19 period was observed. Multivariable analysis identified age, BMI, preoperative Hb, sex, procedure type, IVFCM administration, and transfusion as predictors of postoperative day-4 Hb, while age, IVFCM administration, transfusion, procedure type, and study period were associated with PRM admission. *Conclusions*: Despite major organizational disruption, perioperative hemoglobin levels and transfusion rates remained stable, while hospital length of stay decreased. These findings may partly reflect strict selection of already optimized patients; however, this strategy is not equivalent to structured PBM and cannot be generalized to an unselected arthroplasty population.

## 1. Introduction

Total hip arthroplasty (THA) and total knee arthroplasty (TKA) are among the most commonly performed orthopaedic elective procedures worldwide, with increasing incidence driven by population ageing [1]. Despite their safety and effectiveness, both procedures are associated with substantial perioperative blood loss, reaching 1000–1500 mL in THA [2,3].

Postoperative anemia is a frequent consequence of these procedures and is associated with increased infection rates, longer hospital stay, circulatory overload, and mortality [4]. To reduce anemia-related complications, multidisciplinary patient blood management (PBM) was implemented. Patient registries showed that PBM strategies effectively maintained hemoglobin (Hb) levels, reduced perioperative blood loss, and improved tolerance to anemia through iron deficiency management, resulting in fewer allogeneic blood transfusions [5]. These PBM strategies were introduced at our institution in 2014 and have been adopted as standard care since 2016. All candidates for elective joint replacement underwent multidisciplinary PBM assessment approximately 30 days before surgery, including screening and treatment of anemia and iron deficiency [6]. This approach has been shown to reduce transfusion rates and hospital length of stay in patients undergoing primary THA and TKA [6].

In 2020, the COVID-19 pandemic disrupted this clinical pathway. Government restrictions led to a global reduction in blood donations and constrained blood supplies [7,8,9]. Simultaneously, elective surgeries were postponed as hospitals prioritized COVID-19 care, resulting in a marked reduction in procedures such as THA and TKA [10,11,12,13]. At our institution, this resulted in a substantial change from the standard pre-pandemic pathway. During the COVID-19 period, this structured preoperative optimization pathway was suspended. Moreover, elective surgery was restricted to selected patients considered to be at lower perioperative risk, particularly those aged <80 years, with preoperative hemoglobin > 13.0 g/dL, and without major clinical contraindications to surgery. In parallel, a fast-track model was adopted, with admission on the day of surgery or the day before, reduced preoperative hospital access, and discharge planning aimed at minimizing length of stay. This affected structured preoperative optimization pathways and anemia and iron-deficiency correction.

We therefore hypothesized that disruption of the established PBM pathway during the COVID-19 period would be associated with increased transfusion requirements and worse perioperative outcomes in patients undergoing joint replacement surgery.

This single-center retrospective cohort study aimed to assess the impact of COVID-19-related organizational disruption on the implementation of an established patient blood management (PBM) protocol in primary total hip and knee arthroplasty. This analysis was conducted after the acute pandemic phase to assess the medium-term impact of a major organizational disruption on PBM implementation and related perioperative pathways.

## 2. Materials and Methods

### 2.1. Study Population

This monocentric retrospective cohort study included consecutive patients undergoing primary unilateral total hip arthroplasty (THA) or total knee arthroplasty (TKA) between 2019 and 2023. Patients were stratified into three periods: pre-COVID-19 (January–December 2019), during COVID-19 (September 2020–August 2021), and post-COVID-19 (March 2022–February 2023). January–August 2020 was excluded because elective arthroplasty activity was suspended or negligible during the early pandemic. September 2021–February 2022 was excluded as a transitional reorganisation phase to allow comparison among three clearly defined organisational periods. The study includes patients who underwent surgery between 2019 and 2023; therefore, the available follow-up duration varied according to the date of surgery.

All patients provided informed consent for the use of anonymized data. The study was approved by the Internal Review Board of IRCCS Humanitas Research Center (ID No. 618/17). The ethics approval obtained in December 2017 was issued as a broad authorization by our Institutional Review Board to retrospectively review all patients undergoing total joint arthroplasty at our institution. This approval is not limited to a specific dataset or time period but is designed to cover ongoing and future retrospective analyses within this patient population, as our institution is recognized as a Scientific Institute for Research, Hospitalization and Healthcare (IRCSS). Accordingly, the data included in the present study (collected between 2019 and 2023) fall within the scope of this pre-existing approval.

During the COVID-19 period, resource limitations prevented full implementation of the institutional patient blood management (PBM) protocol. Patients were therefore selected daily by an institutional Emo-Board, which also allocated blood units, prioritizing COVID-19 patients. Eligibility criteria included age < 80 years and preoperative hemoglobin (Hb) > 13.0 g/dL. Only patients deemed fit for surgery were selected to minimize transfusion requirements and postoperative complications. These eligibility criteria created a selected COVID-19 cohort that differed structurally from the pre- and post-COVID-19 cohorts, in which the same restrictions were not applied. Therefore, direct comparability among study periods is inherently limited, and findings from the COVID-19 period may not be generalizable to the broader arthroplasty population.

Before and after the COVID-19 period, the standardized PBM protocol was fully applied, comprising preoperative, intraoperative, and postoperative phases.

### 2.2. PBM Protocol

This PBM protocol was applied for both primary THA and TKA. Patients underwent multidisciplinary assessment at least 30 days before surgery. Estimated surgical blood loss and cardiopulmonary compensation were evaluated. Iron status was assessed using ferritin, C-reactive protein, and transferrin saturation. In patients with iron deficiency (ferritin < 100 μg/L), surgery was postponed and a 1 g dose of intravenous ferric carboxymaltose (IVFCM) was administered; when intravenous treatment was not feasible, patients received oral sucrosomial iron (30 mg) combined with vitamin C (70 mg) for 30 days. Non-steroidal anti-inflammatory drugs were discontinued before surgery. Thromboembolic risk was assessed using the Caprini score [14].

Neuraxial regional anaesthesia with controlled hypotension (mean arterial pressure > 60 mmHg) was preferred [15,16]. Preoperative antibiotic prophylaxis consisted of intravenous cefazolin (2 g) or clindamycin (600 mg in case of β-lactam allergy. Normothermia was maintained intraoperatively and postoperatively. Tranexamic acid (TXA) was routinely administered intravenously with a loading dose of 15 mg/kg approximately 20 min before skin incision, followed by repeated dosing every 8 h during the first 24 postoperative hours. Intraoperative blood salvage was employed whenever blood loss exceeded 10% of total blood volume. Patients were monitored in recovery for 60 min and mobilized approximately 4 h postoperatively, with joint mobility exercises and ambulation using crutches with tolerated weight-bearing for the first postoperative month. All patients received a standardized perioperative medication regimen including intravenous dexamethasone, proton pump inhibitor therapy, antiemetics, and prokinetic agents. Postoperative pain was managed using a multimodal analgesic protocol without continuous regional blocks or patient-controlled analgesia. All patients received postoperative antithromboembolic prophylaxis for a month. A restrictive transfusion strategy was adopted, with red blood cell transfusion considered for Hb < 8 g/dL and guided by clinical status [17,18,19]. Patients were discharged if they were clinically stable, able to ambulate independently with aids, had pain adequately controlled with oral analgesics, and showed no acute complications; otherwise, after four postoperative days they were transferred to the Physical and Rehabilitation Medicine (PRM) department as per institutional protocol.

### 2.3. Outcomes

Demographic variables, including age, sex, body mass index (BMI), and type of procedure (THA or TKA), were collected. Preoperative characteristics included administration of intravenous ferric carboxymaltose (IVFCM), preoperative ferritin levels, and preoperative hemoglobin (Hb) concentration measured on the day of surgical admission. The primary outcomes were transfusion rate, red blood cell (RBC) allocation, transfer to the Physical and Rehabilitation Medicine (PRM) department, hospital length of stay (LOS), and Hb level on postoperative day 4. LOS was defined as the number of days from surgical admission to final hospital discharge, regardless of whether discharge occurred directly from the orthopedic ward or after transfer to the PRM department. RBC allocation was defined as the number of RBC units reserved or assigned preoperatively by the hospital blood bank for perioperative availability according to institutional procedures. The secondary outcomes were factors associated with Hb level on postoperative day 4 and factors associated with PRM admission, assessed through regression analyses. All outcomes were compared across the pre-COVID-19, COVID-19, and post-COVID-19 periods to evaluate the impact of SARS-CoV-2-related organizational disruption on PBM implementation and related perioperative outcomes. THA and TKA were analyzed within the same overall arthroplasty cohort because both procedures were performed within the same institutional elective arthroplasty pathway and managed according to the same PBM protocol, including preoperative anemia screening, transfusion thresholds, and perioperative blood-sparing strategies. However, procedure type was entered as an independent covariate in both univariable and multivariable analyses to account for differences between THA and TKA in blood loss profile and rehabilitation requirements.

### 2.4. Statistical Analysis

Continuous variables were assessed for normality using the Shapiro–Wilk test and are reported as mean ± standard deviation or median and interquartile range, as appropriate. Categorical variables are presented as absolute numbers and percentages. Comparisons among the three study periods were performed using one-way analysis of variance or the Kruskal–Wallis test for continuous variables, and the chi-square test for categorical variables.

Univariable analyses were first performed to explore associations between candidate predictors and the outcomes of interest, namely postoperative day-4 Hb levels and PRM admission. Candidate covariates were selected based on clinical relevance, previous evidence regarding perioperative blood management and rehabilitation outcomes, and availability in the retrospective dataset. These included age, sex, BMI, type of procedure, study period, preoperative Hb, IVFCM administration, and transfusion status. The same prespecified covariates were entered into the multivariable models to ensure consistency and avoid data-driven variable selection. A multivariable linear regression model was used to identify factors independently associated with Hb level on postoperative day 4, entered as a continuous dependent variable. A multivariable logistic regression model was used to identify factors independently associated with PRM admission, entered as a binary dependent variable. Results from linear regression are reported as beta coefficients with 95% confidence intervals, whereas logistic regression results are reported as odds ratios with 95% confidence intervals.

All statistical tests were two-tailed, with a *p* value < 0.05 considered statistically significant. Analyses were conducted using IBM SPSS Statistics for Windows, Version 31.0 (IBM Corp., Armonk, NY, USA).

Missing data were assessed for all variables included in the analyses. Patients with missing values for key outcome variables or covariates included in each regression model were excluded only from the corresponding analysis using a complete-case approach. No imputation was performed. Given the retrospective design, no a priori sample size calculation was conducted; therefore, the analyses should be considered exploratory.

## 3. Results

A total of 5789 patients who underwent primary unilateral total knee arthroplasty (TKA) or total hip arthroplasty (THA) were included in the study. Of these, 1889 procedures were performed in the pre-COVID-19 period, 1416 during COVID-19, and 2484 in the post-COVID-19 period. The mean age was 64.8 ± 11.8 years in the pre-COVID-19 cohort, 64.5 ± 11.7 years during COVID-19, and 66.4 ± 11.1 years post-COVID-19. A slight predominance of female patients was observed in all three groups. Body mass index (BMI) was comparable across cohorts, with median values indicating an overall overweight population. THA was the most frequently performed procedure across all three time periods. Patient demographics are summarized in Table 1.

Baseline patient characteristics, including hemoglobin (Hb) and ferritin levels, were analyzed (Table 1). No significant differences were observed in Hb values on postoperative day 4 (10.9 ± 1.5 g/dL, 10.9 ± 1.4 g/dL, and 10.8 ± 1.4 g/dL) (Table 2). In univariable analysis, neither the COVID-19 period nor the post-COVID-19 period differed significantly from 2019 (*p* = 0.242 and *p* = 0.443, respectively). RBC allocation increased during and after the COVID-19 period, from 105 cases in 2019 to 156 during COVID-19 and 320 post-COVID-19, corresponding to 5.56%, 11.02%, and 12.88% of patients, respectively. Median LOS showed a descriptive reduction across periods, decreasing from 6 days in both the pre-COVID-19 (range 3–50) and COVID-19 (range 3–18) periods to 5 days post-COVID-19 (range 2–28). Transfusion rates were similar across the three periods, with no statistically significant differences observed (Table 2). A reduction was observed in the percentage of patients transferred to Physical and Rehabilitation Medicine (PRM) department, from 25.2% in 2019 to 10.81% during COVID-19 and 7.73% post-COVID-19. In univariable logistic regression, the likelihood of PRM transfer was significantly lower during the COVID-19 period than in 2019 (OR 0.36, 95% CI 0.30–0.44; *p* < 0.001) and decreased further in the post-COVID-19 period (OR 0.24, 95% CI 0.20–0.29; *p* < 0.001).

Multivariable analysis showed that hemoglobin (Hb) levels on postoperative day 4 were positively associated with higher body mass index, higher preoperative Hb levels, and increasing age, and were significantly higher in male patients compared with females, in patients undergoing total knee arthroplasty (TKA) compared with total hip arthroplasty (THA), and in those receiving intravenous ferric carboxymaltose (Table 3). Conversely, postoperative day 4 Hb levels were significantly lower in patients who received blood transfusions (Table 3).

The probability of admission to the Physical and Rehabilitation Medicine (PRM) department increased with advancing age, administration of intravenous ferric carboxymaltose, and receipt of blood transfusion, and decreased in patients undergoing TKA compared with THA (Table 4). Compared with the pre-COVID-19 period (2019), the likelihood of PRM admission was significantly reduced in all subsequent years, with a progressive decrease over time (Table 4).

## 4. Discussion

The principal finding of this study is that postoperative hemoglobin levels and transfusion rates remained stable across the pre-COVID-19, COVID-19, and post-COVID-19 periods, despite major disruption to hospital organization and reduced implementation of the institutional PBM protocol during the pandemic. In contrast, RBC allocation increased during and after the COVID-19 period, while PRM transfers decreased.

During the COVID-19 period, the inability to fully apply the institutional PBM protocol necessitated the introduction of patient selection criteria [20,21]. Only patients with higher preoperative hemoglobin levels and favorable clinical profiles were admitted for elective surgery, with the explicit aim of minimizing transfusion requirements and postoperative complications in the context of limited blood availability. This selection strategy excluded higher-risk patients, likely acting as a surrogate for the preoperative optimization usually achieved through PBM. Indeed, patients undergoing surgery between September 2020 and August 2021 showed preoperative hemoglobin values comparable to those observed in 2019, indicating that they could have been already optimized at baseline. These patient selection criteria were not applied in the pre- or post-COVID-19 periods and therefore represent a major structural difference between cohorts, limiting direct comparability. Accordingly, similar hemoglobin levels and transfusion rates during COVID-19 should not be interpreted as evidence that PBM can be replaced by patient selection. Rather, it suggests that selecting patients who were already clinically and hematologically optimized may have reproduced some measurable effects usually targeted by PBM, particularly transfusion avoidance. However, unlike structured PBM, which actively identifies and treats modifiable risk factors such as iron deficiency and anemia, the COVID-era strategy excluded higher-risk patients; therefore, this interpretation remains hypothesis-generating and cannot be extrapolated to an unselected arthroplasty population. The post-COVID-19 data, showing stable hemoglobin levels and transfusion rates despite increased RBC allocation, further suggest that broader organizational factors influenced blood unit allocation, while restrictive transfusion policies remained effective.

A descriptive reduction in median length of stay was observed in the post-COVID-19 phase. This was accompanied by a decrease in the proportion of patients transferred to Physical and Rehabilitation Medicine departments. This change likely reflects modifications in discharge planning and rehabilitation pathways during and after the pandemic, although the available data do not allow us to determine the relative contribution of clinical factors or organizational changes in rehabilitation access [22]. While a shorter hospitalization period is generally desirable, reduced access to inpatient rehabilitation may disproportionately affect older or more vulnerable patients, and its impact on functional recovery warrants careful consideration. Importantly, multivariable analysis provides further insight into the clinical implications of these organizational changes. The probability of transfer to the PRM department was independently associated with advancing age, blood transfusion, and receipt of intravenous ferric carboxymaltose. In this analysis, IVFCM identifies patients with preoperative iron deficiency requiring PBM intervention and therefore likely represents a proxy for baseline anemia risk or clinical vulnerability, rather than an independent causal determinant of postoperative hemoglobin levels or PRM admission. These findings suggest that reduced access to rehabilitation during the post-COVID-19 period could have affected primarily lower-risk patients, whereas older and more clinically complex individuals may have remained more likely to require structured inpatient rehabilitation.

From a health-economic perspective, PBM has been shown to improve cost-effectiveness by reducing blood product utilization and shortening hospital length of stay [23,24]. In this context, our findings suggest that structured PBM implementation and the selective admission of optimized patients could contribute to a more efficient use of healthcare resources. However, because no formal cost analysis or functional follow-up was performed, potential downstream costs related to outpatient care or delayed recovery should be considered [25].

This study has several strengths. First, it includes a large cohort of more than 5700 patients, providing robust statistical power and enhancing the reliability of the findings. Second, the analysis offers valuable real-world insight into how a major organizational crisis affected the clinical application of patient blood management strategies in elective joint arthroplasty. The interpretation of results is deliberately balanced, avoiding overstatement and acknowledging the influence of pandemic-related patient selection and system constraints.

Several limitations must be acknowledged. First, the retrospective, single-center design limits the generalizability of the findings. Most importantly, the COVID-19 cohort was subject to stricter eligibility criteria, including age < 80 years and preoperative Hb > 13.0 g/dL, which were not applied in the pre- or post-COVID-19 periods. This structural difference introduces substantial selection bias and limits direct comparability among cohorts. Consequently, the observation that COVID-era patient selection may have reproduced some effects of PBM should be interpreted as hypothesis-generating rather than as a formally demonstrated equivalence between patient selection and PBM. In addition, important clinical confounders, including comorbidity burden, ASA status, and perioperative anticoagulant use, were not systematically available and therefore could not be included in the multivariable models. This may have resulted in residual confounding and limits causal interpretation of the observed associations. Moreover, because of the retrospective design, no a priori sample size calculation was performed, and the analyses should be considered exploratory. Functional outcomes and long-term follow-up were unavailable, preventing assessment of the broader clinical impact of reduced rehabilitation access and shortened hospitalization. Finally, although intraoperative PBM measures such as tranexamic acid administration and intraoperative blood salvage were included in the institutional protocol, they were not specifically analyzed in the present study. Data on estimated intraoperative blood loss and the actual rate of blood salvage use were not available; therefore, the independent contribution of these intraoperative strategies to postoperative hemoglobin levels and transfusion rates could not be assessed.

The main message of this study is the distinction between active PBM-driven optimization and crisis-driven selection of already optimized patients. Although both approaches were associated with stable short-term hemoglobin and transfusion outcomes, they are not equivalent in mechanism or generalizability: PBM identifies and treats modifiable risk factors, whereas pandemic-era selection restricted surgery to lower-risk patients. This distinction is relevant for future organizational crises, because preservation of short-term outcomes may occur at the cost of reduced generalizability and limited access for higher-risk patients.

## 5. Conclusions

In conclusion, the SARS-CoV-2 pandemic altered the organization and delivery of elective joint replacement surgery, limiting the application of established PBM protocols. Nevertheless, despite reduced implementation of the established PBM protocol during the COVID-19 period, postoperative day-4 hemoglobin levels and transfusion rates remained stable across the pre-COVID-19, COVID-19, and post-COVID-19 periods. These findings suggest that strict selection of already optimized patients may have contributed to the preservation of short-term perioperative blood management outcomes during a period of major organizational disruption; however, this strategy should not be considered equivalent to structured PBM and cannot be generalized to an unselected arthroplasty population.

## Figures and Tables

**Table 1:** Patient demographics and pre-operative values (BMI, body mass index; IVFCM, intravenous ferric carboxymaltose; TKA, total knee arthroplasty; RBCs, red blood cells).

	Pre-COVID-19	COVID-19	Post-COVID-19
**Number of surgeries**	1889	1416	2484
**Age (years)**	64.8 ± 11.8	64.5 ± 11.7	66.4 ± 11.1
**Sex (M)**	813 (43.04%)	668 (47.18%)	1105 (44.48%)
**BMI (kg/m^2^)**	27.8 ± 4.7	27.8 ± 4.3	27.9 ± 4.6
**IVFCM**	353 (18.69%)	88 (6.21%)	468 (18.84%)
**Surgery (TKA)**	658 (34.83%)	549 (38.77%)	944 (38.00%)
**Preoperative Ferritin (μg/L)**	79.6 (range 3–1606)	88.3 (range 3.1–1983)	82.5 (range 3.5–1030.5)
**Preoperative Hb (g/dL)**	14.2 ± 1.3	14.3 ± 1.3	13.9 ± 1.3
**Allocated RBCs units**	105 (5.56%)	156 (11.02%)	320 (12.88%)

**Table 2:** Perioperative and postoperative values (PRM, Physical and Rehabilitation Medicine; LOS, length of stay, POD, postoperative day).

	Pre-COVID-19	COVID-19	Post-COVID-19
**Transfusions**	88 (4.66%)	70 (4.94%) (*p* = 0.261)	110 (4.43%) (*p* = 0.512)
**PRM transfer**	476 (25.20%)	153 (10.81%)	192 (7.73%)
**LOS (days)**	6 (range 3–50)	6 (range 3–18)	5 (range 2–28)
**Hb POD 4 (g/dL)**	10.9 ± 1.5	10.9 ± 1.4 (*p* = 0.242)	10.8 ± 1.4 (*p* = 0.443)

**Table 3:** Univariable and multivariable linear regression analysis for factors associated with hemoglobin on postoperative day 4 (Hb POD 4).

Hb POD 4	Univariable		Multivariable	
	**Coeff (95%CI)**	***p***	**Coeff (95%CI)**	***p***
**IVFCM**	−0.66 (−0.75; −0.56)	<0.001	−0.66 (−0.75; −0.56)	<0.001
**Sex**	0.71 (0.64; 0.77)	<0.001	0.71 (0.64; 0.77)	<0.001
**Age**	0.06 (0.05; 0.07)	<0.001	0.06 (0.05; 0.07)	<0.001
**BMI**	−0.01 (−0.01; −0.00)	<0.001	−0.01 (−0.01; −0.00)	<0.001
**Preoperative Hb**	0.56 (0.54; 0.58)	<0.001	0.56 (0.54; 0.58)	<0.001
**Transfusions**	−2.38 (−2.53; −2.23)	<0.001	−2.38 (−2.53; −2.23)	<0.001
**TKA**	0.59 (0.52; 0.65)	<0.001	0.59 (0.52; 0.65)	<0.001
**Pre-COVID-19**	0			
**COVID-19**	0.06 (−0.04; 0.16)	0.242		
**Post-COVID-19**	−0.03 (−0.12; 0.05)	0.443		

**Table 4:** Univariable and multivariable linear regression analysis for factors associated with admission to the PRM department.

PRM Transfer	Univariable		Multivariable	
	OR (95%CI)	*p*	Coeff (95%CI)	*p*
**IVFCM**	1.56 (1.31; 1.87)	<0.001	1.32 (1.09; 1.60)	0.005
**Sex (M)**	0.75 (0.65; 0.87)	<0.001	0.79 (0.67; 0.91)	0.002
**Age**	1.01 (1.01; 1.02)	<0.001	1.01 (1.01; 1.02)	<0.001
**BMI**	0.99 (0.98; 1.01)	0.387	-	
**Preoperative Hb**	0.86 (0.82; 0.91)	<0.001	-	
**Transfusions**	3.52 (2.77; 4.49)	<0.001	3.28 (2.53; 4.24)	<0.001
**TKA**	0.59 (0.51; 0.69)	<0.001	0.55 (0.46; 0.64)	<0.001
**Pre-COVID-19**	1		1	
**COVID-19**	0.36 (0.30; 0.44)	<0.001	0.37 (0.30; 0.45)	<0.001
**Post-COVID-19**	0.24 (0.20; 0.29)	<0.001	0.23 (0.19; 0.28)	<0.001

## Data Availability

The original contributions presented in this study are included in the article. Further inquiries can be directed to the corresponding author.
